# Empagliflozin Induces White Adipocyte Browning and Modulates Mitochondrial Dynamics in KK Cg-Ay/J Mice and Mouse Adipocytes

**DOI:** 10.3389/fphys.2021.745058

**Published:** 2021-10-27

**Authors:** Linxin Xu, Chaofei Xu, Xiangyang Liu, Xiaoyu Li, Ting Li, Xiaochen Yu, Mei Xue, Jing Yang, Constantine E. Kosmas, Dimitrios Moris, Fabian Sanchis-Gomar, Naofumi Yoshida, Nathan A. Berger, Wilbert S. Aronow, Bei Sun, Liming Chen

**Affiliations:** ^1^NHC Key Laboratory of Hormones and Development, Tianjin Key Laboratory of Metabolic Diseases, Tianjin Medical University Chu Hsien-I Memorial Hospital & Tianjin Institute of Endocrinology, Tianjin, China; ^2^Department of Endocrinology, The First Hospital of Shanxi Medical University, Shanxi Medical University, Taiyuan, China; ^3^Department of Medicine, Montefiore Medical Center, Bronx, NY, United States; ^4^Department of Surgery, Duke University Medical Center, Durham, NC, United States; ^5^Department of Physiology, Faculty of Medicine, University of Valencia and INCLIVA Biomedical Research Institute, Valencia, Spain; ^6^Division of Cardiovascular Medicine, Department of Internal Medicine, Kobe University Graduate School of Medicine, Kobe, Japan; ^7^Department Biochemistry, Genetics & Genome Sciences, Case Comprehensive Cancer Center, Case Western Reserve University School of Medicine, Cleveland, OH, United States; ^8^Cardiology Department, School of Medicine, Westchester Medical Center and New York Medical College, Valhalla, NY, United States

**Keywords:** browning, mitochondria, fusion, mitochondrial dynamics, type 2 diabetes mellitus, sodium-glucose co-transporter-2 inhibitor

## Abstract

**Background:** White adipose tissue (WAT) browning is a promising target for obesity prevention and treatment. Empagliflozin has emerged as an agent with weight-loss potential in clinical and *in vivo* studies, but the mechanisms underlying its effect are not fully understood. Here, we investigated whether empagliflozin could induce WAT browning and mitochondrial alterations in KK Cg-Ay/J (KKAy) mice, and explored the mechanisms of its effects.

**Methods:** Eight-week-old male KKAy mice were administered empagliflozin or saline for 8 weeks and compared with control C57BL/6J mice. Mature 3T3-L1 adipocytes were treated in the presence or absence of empagliflozin. Mitochondrial biosynthesis, dynamics, and function were evaluated by gene expression analyses, fluorescence microscopy, and enzymatic assays. The roles of adenosine monophosphate–activated protein kinase (AMPK) and peroxisome proliferator–activated receptor-γ coactivator-1-alpha (PGC-1α) were determined through AICAR (5-Aminoimidazole-4-carboxamide1-β-D-ribofuranoside)/Compound C and RNA interference, respectively.

**Results:** Empagliflozin substantially reduced the bodyweight of KKAy mice. Mice treated with empagliflozin exhibited elevated cold-induced thermogenesis and higher expression levels of uncoupling protein 1 (UCP1) and other brown adipose tissue signature proteins in epididymal and perirenal WAT, which was an indication of browning in these WAT depots. At the same time, empagliflozin enhanced fusion protein mitofusin 2 (MFN2) expression, while decreasing the levels of the fission marker phosphorylated dynamin-related protein 1 (Ser616) [p-DRP1 (Ser616)] in epididymal and perirenal WAT. Empagliflozin also increased mitochondrial biogenesis and fusion, improved mitochondrial integrity and function, and promoted browning of 3T3-L1 adipocytes. Further, we found that AMPK signaling activity played an indispensable role in empagliflozin-induced browning and mitochondrial biogenesis, and that PGC-1α was required for empagliflozin-induced fusion. Whether empagliflozin activates AMPK by inhibition of SGLT2 or by independent mechanisms remains to be tested.

**Conclusion:** Our results suggest that empagliflozin is a promising anti-obesity treatment, which can immediately induce WAT browning mitochondrial biogenesis, and regulate mitochondrial dynamics.

## Introduction

Obesity is a growing threat to public health due to its association with various metabolic diseases. The rise in obesity has prompted the investigation of novel effective treatments to combat the condition (Pilitsi et al., 2019). Obesity arises due to excess energy storage relative to energy expenditure in adipocytes. Therefore, restoring energy homeostasis by promoting energy expenditure is a viable strategy for treating obesity (Roden and Shulman, 2019).

Two established types of adipose tissue are present in mammals: white and brown adipose tissue (WAT and BAT, respectively) (Frontini and Cinti, 2010). They can be distinguished from each other by their morphology, biochemical characteristics, and function. BAT function is mediated by uncoupling protein 1 (UCP1). Localized in the inner mitochondrial membrane, UCP1 uncouples respiration from adenosine triphosphate synthesis to generate heat (Pilitsi et al., 2019). Although the formation of BAT in humans is believed to be completed before birth, emerging evidence has shown that beige or “brite” (brown in white) cells, a subset of WAT cells, can be converted to BAT-like adipocytes in response to specific environmental cues (Wu et al., 2012; Frontini et al., 2013). In consequence, WAT browning has attracted much attention as a therapeutic tool to enhance energy expenditure and treat obesity.

At a cellular level, nutrient metabolism and energy production are mainly accomplished by mitochondria. These organelles undergo dynamic processes of biogenesis, degradation, fusion, and fission. Active mitochondrial biogenesis leads to an increase in mitochondrial volume density, which is observed in BAT and during WAT browning (Ikeda et al., 2018). Peroxisome proliferator–activated receptor (PPAR)-γ coactivator-1-alpha (PGC-1α), a protein highly expressed in BAT, positively regulates mitochondrial biogenesis through the coactivation of downstream transcription factors such as nuclear factor erythroid 2-related factor 2 (NRF2) and mitochondrial transcription factor A (TFAM) (Arany et al., 2005). Mitochondrial morphology and quality are controlled by mitochondrial fusion and fission (Gomar and Derbré, 2014). While fusion is associated with long and integral mitochondria, fission generates small and depolarized ones. Fusion is regulated by mitofusins (MFN1 and MFN2), among which MFN2 is commonly found in mammalian tissues and is highly expressed in mitochondria-rich tissues, whereas fission is controlled by dynamin-related protein 1 (DRP1) (Bach et al., 2003; Chen and Chan, 2005). The disruption of mitochondrial biogenesis and dynamics is relevant to the pathogenesis of metabolic diseases. For instance, Heinonen et al. (2015) reported that mitochondrial biogenesis was downregulated in individuals with obesity, and was associated with a lower number and reduced metabolic activity of mitochondria. Decreased mitochondrial fusion and increased fission have also been observed in patients with comorbid obesity and type 2 diabetes (T2DM). Obesity and T2DM are related to mitochondrial dysfunction and increased reactive oxygen species production (Shenouda et al., 2011; de Mello et al., 2018).

Sodium-glucose co-transporter-2 (SGLT2, also called solute carrier family 5 [sodium/glucose cotransporter], member 2, SLC5A2) inhibitors are established as effective treatments for T2DM. They decrease blood glucose by reducing its renal reabsorption and increasing its excretion in urine (Steven et al., 2017). Empagliflozin is an SGLT2 inhibitor approved for the treatment of T2DM, which can reduce the risk of cardiovascular death in patients with T2DM and cardiovascular disease (JARDIANCE prescribing information, 2020). Clinical trials have found empagliflozin to be associated with weight loss in patients with T2DM (Häring et al., 2013, 2014; Rosenstock et al., 2014). Empagliflozin has also been shown to promote weight loss in animal models, which might be attributed to the enhanced expression of UCP1 in BAT and WAT, and increased energy expenditure (Xu et al., 2017, 2019). However, the mechanism by which empagliflozin induces these metabolic changes and weight loss requires further investigation.

The present study aimed to provide *in vivo* and *in vitro* evidence to elucidate the possible mechanisms of empagliflozin’s weight-loss effect. We hypothesized that empagliflozin might control or reduce bodyweight via inducing WAT browning. We tested this hypothesis in an *in vivo* mouse model of obesity. Alterations in mitochondrial biogenesis and dynamics in relation to WAT browning under empagliflozin treatment were further studied *in vivo* and *in vitro*. Finally, the mechanisms underlying empagliflozin-induced adipocyte browning and mitochondrial modulation were explored.

## Materials and Methods

### Animal Studies

Male KK Cg-Ay/J (KKAy) mice, which are homozygous for the yellow spontaneous mutation. they were originally developed by Japanese scholars, who transferred the Ay gene into KK mice (Iwatsuka et al., 1970) and present with glucose intolerance, insulin resistance, and obesity, were used as an animal model in this study. C57BL/6J mice were used as a healthy control group. Seven-week-old male KKAy mice and male C57BL/6J mice were purchased from HFK Bioscience (Beijing, China). The mice were kept under specific-pathogen-free conditions in environmentally controlled clean rooms at 22°C under a 12/12-h light/dark cycle in the animal facility at Tianjin Medical University (Tianjin, China). The KKAy mice were fed a high-fat diet (consisting of 17.9% fat, 48% carbohydrate, and 17.5% protein), and the C57BL/6J mice were fed a chow diet (regular chow comprising 5% fat, 53% carbohydrate, and 23% protein).

After acclimatization for 1 week, the KKAy mice were randomly divided into two groups (*n* = 6 in each group) and treated with saline or empagliflozin for 8 weeks. Empagliflozin was administered via oral gavage at a dose of 3.8 mg/kg/day with 0.5% hydroxyethyl cellulose as the carrier. The dosage of Empa in KK-Ay mice was converted from the medicine operation instructions in human (maximum dosage 25 mg/d) according to the body surface area. In experiments to assess the effect of insulin, KKAy mice were administered 0.5 U/kg/day of insulin for 8 weeks (Actrapid, Novo Nordisk). At the completion of treatment, the mice were fasted overnight and euthanized by exsanguination under anesthesia with inhaled 5% isoflurane in room air. Following euthanasia, the collected adipose weight was measured and adipose tissues were removed for subsequent experiments. All animal experiments and procedures were approved by the Animal Ethics and Experimental Committee of the Metabolic Disease Hospital of Tianjin Medical University.

### Cold Resistance and Intraperitoneal Glucose Tolerance Test

To assess resistance to cold, mice were placed in a 4°C environment for 4 h. Their rectal temperatures were measured between 8 pm and 12 am with a microprobe thermometer (ThermoWorks, Alpine, UT, United States). To assess glucose tolerance, mice were fasted overnight and then administered a glucose solution. Blood samples were taken at 15-min intervals over a 120-min period, and changes in blood glucose were examined.

### Cell Culture and Treatment

The preadipocyte cell line 3T3-L1 was cultured in Dulbecco’s Modified Eagle Medium (DMEM, Institute of Endocrinology of Tianjin Medical University, China) with 10% fetal bovine serum (FBS) and maintained in a humidified incubator with 5% CO2 at 37°C. Adipocyte differentiation was induced by treating preadipocytes with the classical hormone cocktail of insulin (Novorin R, Denmark), indomethacin (Solarbio, United States), 3-isobutyl-1-methyl xanthine (Solarbio, United States), and dexamethasone (Solarbio, United States) for 5 days. Mature 3T3-L1 adipocytes were treated with empagliflozin (4 μmol/L) in normal medium for 3 days. To assess the role of adenosine monophosphate–activated protein kinase (AMPK), mature adipocytes were treated in the presence or absence of empagliflozin, with or without the addition of 5-Aminoimidazole-4-carboxamide ribonucleotide (AICAR; 10 μmol/L, MCE, United States) or Compound C (1 μmol/L, Sigma-Aldrich).

### Hematoxylin Staining, Immunofluorescence, and Immunohistochemistry

White adipose tissue samples were fixed overnight in 4% paraformaldehyde, after which they were sliced into 5-μm-thick sections. Cells grown on coverslips were fixed in 4% paraformaldehyde at room temperature and permeabilized with 0.1% Triton X-100 (Sigma, United States). For histologic examination, tissue slices were stained with hematoxylin. For immunofluorescence and immunohistochemistry, tissue slices or cells were incubated with blocking buffer (1% bovine serum albumin) for 1 h, and then with anti-UCP1 (Proteintech, China), anti-COX IV antibody (Proteintech, China), or anti-MFN2 antibody (Cell Signaling Technology, United States) at 4°C overnight. After that, the tissue slices or cells were incubated with fluorescein isothiocyanate (FITC)–conjugated secondary antibody (for immunofluorescence) or horseradish peroxidase–conjugated secondary antibody (for immunohistochemistry) at room temperature for 1 h. Nuclei were counterstained with 4′,6-diamidino-2-phenylindole (DAPI, Zhongshan Jinqiao, ZLI-9557). Immunofluorescence images were captured with an AV300-ASW confocal microscope (Olympus America Inc., Center Valley, PA, United States).

### Oil Red O Staining

Tissue slices or cells were stained with filtered Oil Red O for 5–10 min (isopropanol:water = 3:2). An optical microscope (Olympus, Japan) was used to obtain images. To quantify the Oil Red O staining, intracellular lipids were extracted with isopropanol and homogenized through 10 min of shaking at room temperature. The absorbance was measured at 520 nm with a monochromator microplate reader (Bio Tek). The data from three independent experiments are presented.

### Small Interfering RNA Transfection

Custom-made *Pgc-1*α stealth small interfering RNA (siRNA) (Sense:5′-GACGACAAAUCAGACAAGATT-3′; antisense 5′-UCUUGUCUGAUUUGUCGUCTT-3′) and negative control (Sense:5′-UUCUCCGAACGUGUCACGUTT-3′; Antisense 5′-ACGUGACACGUUCGGAGAATT-3′) were purchased from GenePharma (Shanghai). According to the manufacturer’s protocol, Lipofectamine 2000 (Invitrogen, 11668019) was used to transfect 3T3-L1 adipocytes with siRNA at a final concentration of 20 mM for 6 h.

### Western Blot Analysis

Proteins were extracted from cells or tissues with RIPA lysis buffer (Solarbio, China) supplemented with phenylmethylsulfonyl fluoride (PMSF) and phosphatase inhibitors (Calyculin A), and then dissolved in sodium dodecyl sulfate (SDS) loading buffer. Equal amounts of protein were resolved by SDS-polyacrylamide gel electrophoresis (SDS–PAGE) and transferred to a polyvinylidene difluoride (PVDF) membrane. After being blocked with 5% skim milk in tris-buffered saline with Tween (0.25%), the membrane was incubated with primary antibodies at 4°C (Supplementary Table 1). Primary antibodies were detected with the corresponding peroxidase-conjugated immunoglobulin G (IgG) antibody. Band densitometry was analyzed using Image J software (National Institutes of Health, Bethesda, MD, United States).

### Quantitative Reverse Transcription–Polymerase Chain Reaction

Messenger RNA (mRNA) levels were assessed by performing quantitative reverse transcription–polymerase chain reaction (qRT-PCR) using a SYBR Green PCR kit (Sangon Biotech, China) on a CFX96 real-time PCR system (Bio-Rad, United States). All primers were synthesized by TSINGKE Biotechnology (Beijing, China). Primer sequences are shown in Supplementary Table 2.

### Extraction, Enzyme Activity, and Function of Mitochondria

Mitochondria were extracted using a mitochondria extraction kit (KeyGEN BioTECH, Nanjing, China) according to the manufacturer’s instructions. Mitochondrial respiratory chain complex I (complex I, NADH-coenzyme Q reductase) and alpha-ketoglutarate dehydrogenase (α-KGDH) activity was detected using quantitative colorimetric assay kits (KGP850/KGP8100, KeyGEN BioTECH, Nanjing, China and C0715, Solarbio, United States) in adherence with the manufacturers’ instructions. The integrity of mitochondrial function was assessed using the cationic dye JC-1 (5,5′, 6,6′-tetrachloro-1,1′, 3,3′-tetraethylaminophthalocyanine, Solarbio, United States), and staining and analysis were carried out in accordance with the manufacturer’s instructions. Mitochondrial morphology was observed by performing MitoTracker staining. Cells cultured on glass slides were incubated with MitoTracker red CMXRos (200 nM) at 37°C for 30 min, which was followed by MFN2 immunofluorescence staining. MitoTracker Red labeled cells were observed under a confocal laser microscope.

### Statistical Analysis

Statistical analyses were performed using Prism 8.0 software (GraphPad, La Jolla, CA, United States). Values are shown as the mean ± standard error of the mean (SEM). Differences among groups were tested by analysis of variance (ANOVA). Statistical significance was indicated by *P* < 0.05.

## Results

### Empagliflozin Reduces Bodyweight and Fat Mass and Enhances Cold-Induced Thermogenesis in KK Cg-Ay/J Mice

Compared with C57BL/6J mice (fed a chow diet), KKAy mice (on a high-fat diet) had increased bodyweight and showed impaired thermogenesis under exposure to cold (Supplementary Figures 1A–D). Hematoxylin staining showed significantly larger lipid droplets in KKAy mice than in C57BL/6J mice (Supplementary Figures 1E,F). Therefore, the subsequent experiments were focused on the effects of empagliflozin in KKAy mice.

**FIGURE 1:**
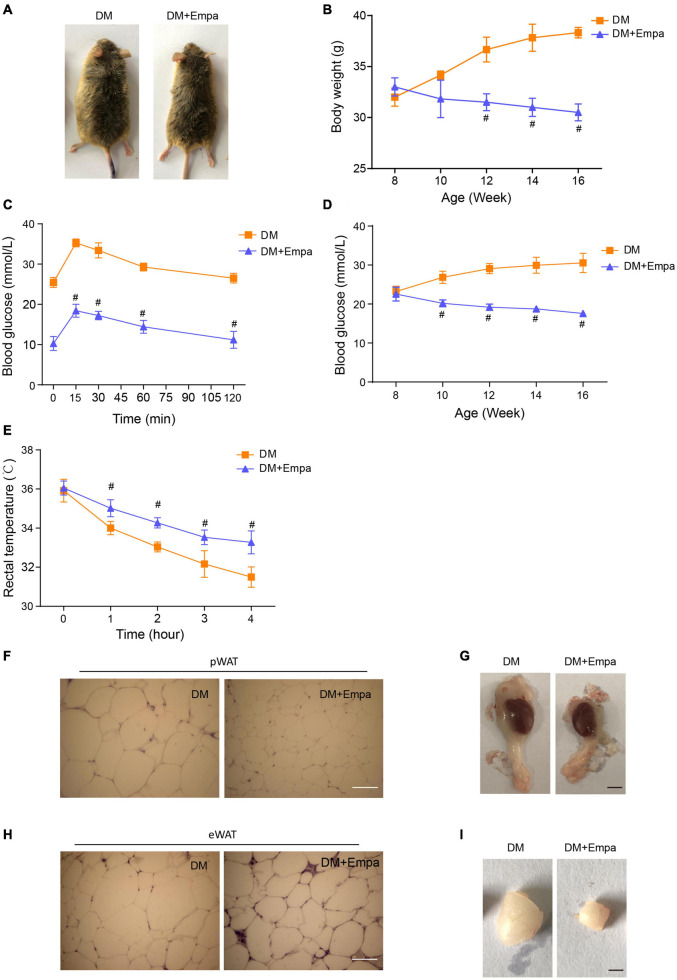
Empagliflozin treatment reduces bodyweight and promotes thermogenesis in KKAy mice. Eight-week-old male KKAy mice (*n* = 6 per group) were administered saline (DM) or empagliflozin (DM + Empa) for 8 weeks. **(A)** Representative images of mice in 2 groups after 8 weeks of treatment; and **(B)** bodyweights of the mice during 8 weeks of treatment. **(C)** Changes in blood glucose as detected by IPGTT after 8 weeks of treatment, and **(D)** postprandial blood glucose levels during 8 weeks of treatment. **(E)** Body temperature during a 4-hour cold test at 4°C. **(F,H)** Representative images of hematoxylin staining, and **(G,I)** representative images of pWAT and eWAT in mice after 8 weeks of treatment. Scale bar represents 100 μm in **(F,H)** and 10 mm in **(G,I)**. Data are presented as the mean ± SEM. ^#^*P* < 0.05 vs. the DM group. pWAT, perirenal white adipose tissue; eWAT, epididymal white adipose tissue. IPGTT, Intraperitoneal glucose tolerance test.

Following the administration of empagliflozin, the bodyweight of KKAy mice decreased gradually in a time-dependent manner, reducing by approximately 25% after 8 weeks of treatment (Figures 1A,B). The hyperglycemia and glucose intolerance were also evidently improved by empagliflozin treatment (Figures 1C,D). The WAT of empagliflozin-treated KKAy mice contained smaller multilocular lipid droplets than that of their untreated counterparts (a 57% decrease in the perirenal WAT (pWAT) of empagliflozin-treated KKAy mice, *P* = 0.018; a 36% decrease in the epididymal WAT (eWAT) of empagliflozin-treated KKAy mice, *P* = 0.024), and these morphological features typical of BAT were most evident in eWAT and pWAT (Supplementary Figures 2A,B, and Figures 1F,H). Therefore, in subsequent experiments, we focused on the effects of empagliflozin on eWAT and pWAT. As well as reducing the lipid content, empagliflozin decreased the size of pWAT and eWAT fat pads in KKAy mice (Figures 1G,I). While KKAy mice showed impaired thermogenesis under cold exposure, empagliflozin treatment enhanced cold-induced thermogenesis (Figure 1E).

Next, we verified the expression of SGLT2, the target protein of empagliflozin, in various organs (the heart, liver, kidney, and brain) of C57BL/6J and KKAy mice (Supplementary Figures 2C–F). SGLT2 expression was found in adipose tissues at different anatomical locations, with the expression in BAT being the highest (Supplementary Figures 2G,H).

### Empagliflozin Upregulates Uncoupling Protein 1 and Other Brown Fat–Related Proteins in Perirenal White Adipose Tissue and Epididymal White Adipose Tissue

**FIGURE 2:**
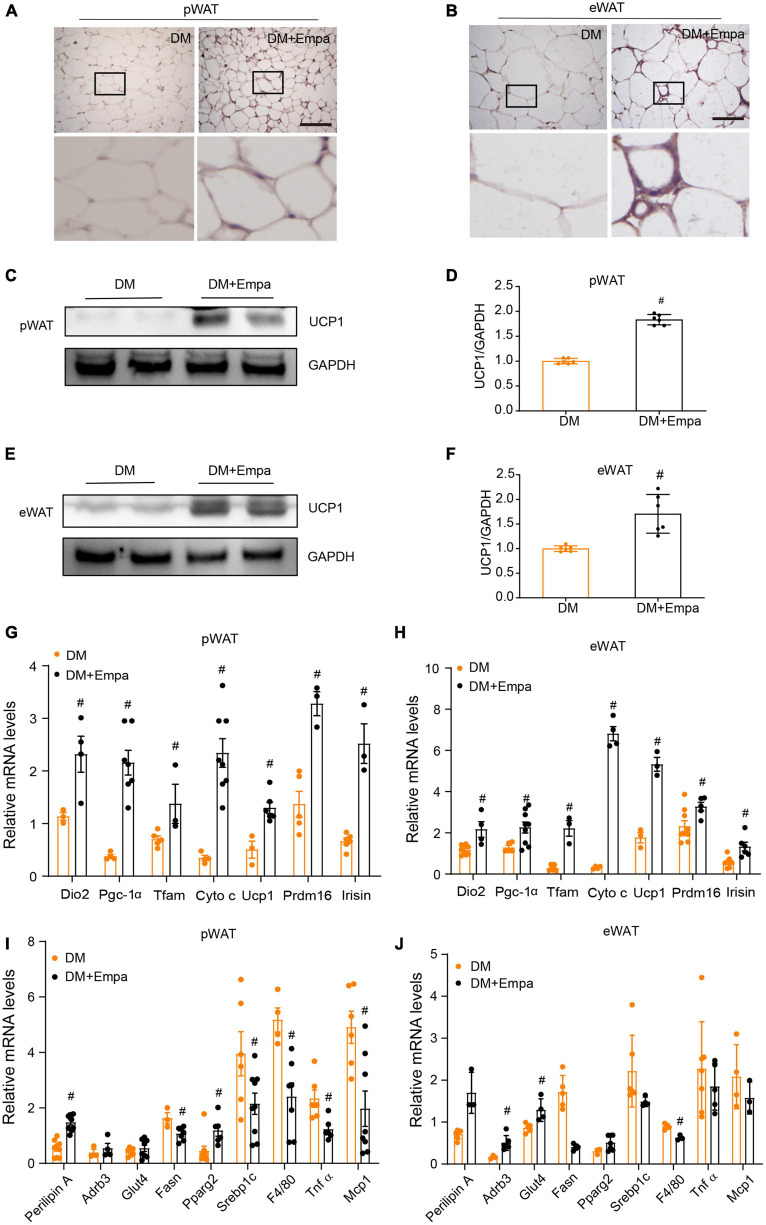
Empagliflozin increases the expression of UCP1 and brown fat-related proteins in the pWAT and eWAT of KKAy mice. Eight-week-old male KKAy mice (*n* = 6 per group) were treated with saline (DM) or empagliflozin (DM + Empa) for 8 weeks. **(A,B)** Immunohistochemical staining of UCP1 in pWAT and eWAT sections. Scale bar represents 100 μm. **(C–F)** Western blot and quantitation of UCP1 expression in pWAT and eWAT, with GAPDH serving as a loading control. **(G–J)** qRT-PCR of mRNAs of *Ucp1* and genes related to mitochondrial biogenesis (*Dio2*, *Pgc-1*α, *Tfam*, and *Cyto c*), thermogenesis (*Prdm16* and *Irisin*), lipolysis (*Plin1*, *Adrb3*, and *Pparg*), glucose metabolism (*Glut4*), lipogenesis (*Fasn* and *Srebf1*), and inflammation (*MCP1*, *Tnf*α, and *F4/80*) in pWAT and eWAT, with GAPDH serving as a loading control. Data are presented as the mean ± SEM. ^#^*P* < 0.05 vs. the DM group. pWAT, perirenal white adipose tissue; eWAT, epididymal white adipose tissue.

The phenotypic changes in KKAy mice treated with empagliflozin prompted us to examine whether empagliflozin could induce WAT browning. Immunohistochemical staining of pWAT and eWAT revealed that the classical BAT marker protein UCP1 was expressed at an indiscernible level in KKAy mice (Figures 2A,B). However, empagliflozin substantially elevated the levels of UCP1 in the pWAT and eWAT of KKAy mice (Figures 2A,B), and this effect was confirmed by western blot (Figures 2C–F). Aside from its effects on *Ucp1*, as detected by qRT-PCR, empagliflozin also upregulated the expression levels of other marker genes characteristic of BAT, such as those involved in thermogenesis (*Prdm16* and *Irisin*) and mitochondrial biogenesis (*Dio2*, *Pgc-1*α, *Tfam*, and *Cyto c*) (Figures 2G,H). Furthermore, the transcriptional profiles of genes involved in lipolysis (*Plin1*, *Adrb3*, and *Pparg*), lipogenesis (*Fasn* and *Srebf1*), and glucose uptake (*Glut4*), as well as those of inflammation-related genes (*MCP1, Tnf*α, and *F4/80*), which were dysregulated in KKAy mice, were restored by empagliflozin treatment (Figures 2I,J). These results suggest that empagliflozin induces browning and adaptive metabolic reprogramming in the pWAT and eWAT of KKAy mice.

### Empagliflozin Enhances Mitochondrial Biogenesis and Respiratory Activity, and Activates Adenosine Monophosphate–Activated Protein Kinase Signaling in Perirenal White Adipose Tissue and Epididymal White Adipose Tissue

**FIGURE 3:**
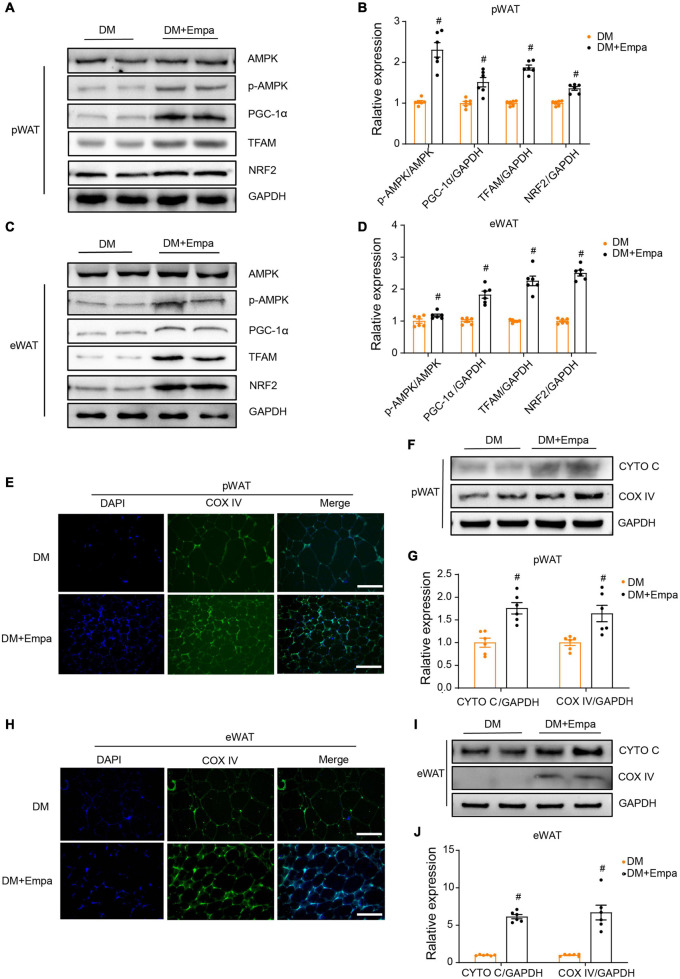
Empagliflozin promotes mitochondrial biogenesis and activates the AMPK signaling pathway in the pWAT and eWAT of KKAy mice. Eight-week-old male KKAy mice (*n* = 6 per group) were treated with saline (DM) or empagliflozin (DM + Empa) for 8 weeks. **(A–D)** Western blot and quantitation of AMPK, p-AMPK, PGC-1α, TFAM, and NRF2 in pWAT and eWAT, with GAPDH serving as a loading control. **(E,H)** Immunofluorescence staining of COX IV (green), with nuclei stained blue with 4′,6-diamidino-2-phenylindole (DAPI). Image magnification is 100 ×. **(F,G,I,J)** Western blot and quantitation of CYTO C and COX IV in pWAT and eWAT, with GAPDH used as a loading control. Data are presented as the mean ± SEM. ^#^*P* < 0.05 vs. the DM group. pWAT, perirenal white adipose tissue; eWAT, epididymal white adipose tissue.

Our preliminary results showed that empagliflozin could induce mitochondrial biogenesis in WAT (Figures 2G,H). AMPK is a key intracellular metabolic sensor that regulates mitochondrial biogenesis via PGC-1α and downstream transcription factors, such as NRF2 and TFAM, to meet energy demand (Scarpulla, 2011). In KKAy mice, the levels of phosphorylated AMPK (p-AMPK), PGC-1α, TFAM, and NRF2 were decreased in both pWAT and eWAT (Figures 3A–D). Importantly, however, empagliflozin treatment reversed the trend of AMPK signaling decline, leading to increases in p-AMPK, PGC-1α, TFAM, and NRF2 (Figures 3A–D). Immunofluorescence revealed that the mitochondrial marker protein COX IV was expressed at a lower level in the pWAT and eWAT of KKAy mice, but it was upregulated by empagliflozin (Figures 3E,H). Correspondingly, western blot showed that mitochondrial biogenesis proteins such as CYTO C and COX IV, the expressions of which were decreased in KKAy mice, were also expressed at a higher level after empagliflozin treatment (Figures 3F,G,I,J). These observations suggest that empagliflozin activates AMPK signaling, which is a plausible upstream regulator of mitochondrial biogenesis.

### Empagliflozin Regulates Mitochondrial Dynamics in Perirenal White Adipose Tissue and Epididymal White Adipose Tissue

**FIGURE 4:**
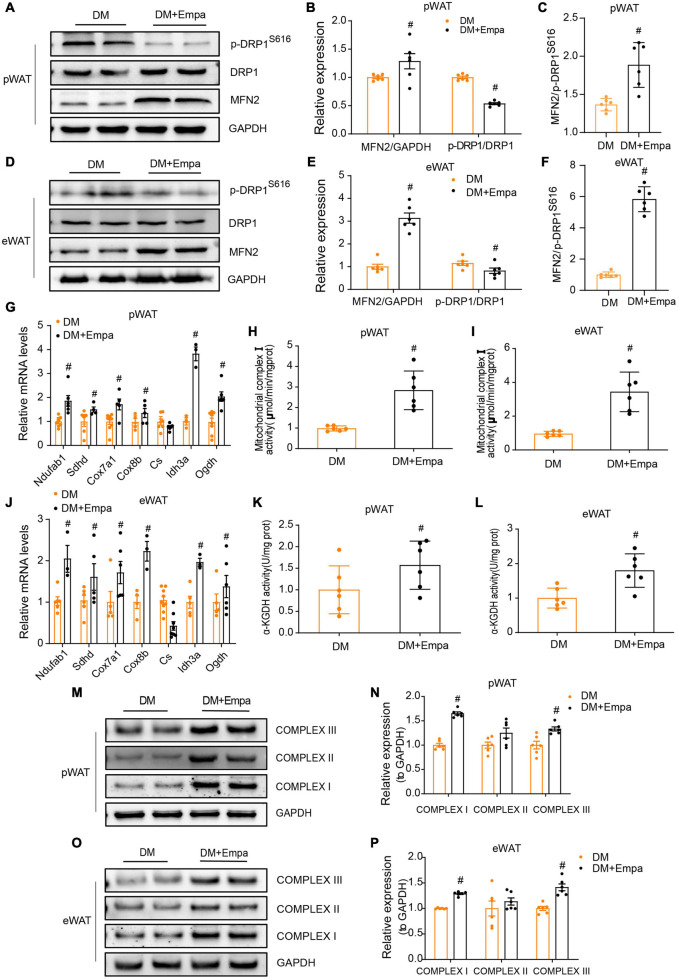
Empagliflozin regulates proteins mediating mitochondrial fusion and fission, and enhances mitochondrial function in the pWAT and eWAT of KKAy mice. Eight-week-old male KKAy mice (*n* = 6 per group) were administered saline (DM) or empagliflozin (DM + Empa) for 8 weeks. **(A–F)** Western blot and quantitation of DRP1, p-DRP1 (S616), and MFN2 in pWAT and eWAT, with GAPDH used as a loading control. **(G,J)** qRT-PCR analysis of mRNAs of complex I (*Ndufab1*), II (*Sdhd*), III (*Cox7a1*), IV (*Cox8b*), and genes involved in the TCA cycle (*Cs*, *Idh3a*, and *Ogdh*) in pWAT and eWAT, with GAPDH serving as a loading control. **(H,I)** Measurement of complex I and **(K,L)** α-KGDH enzymatic activity in pWAT and eWAT, with GAPDH serving as a loading control. **(M–P)** Western blot and quantitation of mitochondrial respiratory chain complex I (NDUFA9), II (SDHA), and III (CYTB) in pWAT and eWAT, with GAPDH used as a loading control. Data are presented as the mean ± SEM. ^#^*P* < 0.05 vs. the DM group. pWAT, perirenal white adipose tissue; eWAT, epididymal white adipose tissue.

The equilibrium in the number and activity of mitochondria is controlled by the dynamic processes of fusion and fission, which are mediated by MFN1/2 and DRP1, respectively (Chen and Chan, 2005). Phosphorylation of DRP1 on Ser616 results in the transport of DRP1 from the cytoplasm to the mitochondrial membrane and the initiation of the fission process (Chang and Blackstone, 2010). In the pWAT and eWAT of KKAy mice, the protein expression of MFN2 was decreased, whereas the levels of p-DRP1 (S616) were increased (Figures 4A–F). However, these changes were reversed by empagliflozin treatment (Figures 4A–F). These results suggest that empagliflozin may induce an alteration in the balance between mitochondrial fusion and fission.

Since mitochondrial fusion is a modulatory mechanism of mitochondrial metabolic activity that is critical for maintaining energy balance, we next evaluated the levels of mitochondrial proteins in the electron transport chain and tricarboxylic acid (TCA) cycle, which are important for energy production in pWAT and eWAT. Through qRT-PCR, we confirmed an empagliflozin-induced increase in the expression of mitochondrial respiratory chain complex proteins and demonstrated that the transcription of genes in the TCA cycle (i.e., *Cs*, *Idh3a*, and *Ogdh*) was also upregulated by empagliflozin (Figures 4G,J). Correspondingly, western blot showed that the mitochondrial respiratory chain complex proteins (i.e., complex I–III), the expressions of which were decreased in KKAy mice, were also expressed at increased levels following empagliflozin treatment (Figures 4M–P).

Next, we measured enzyme activity in the electron transport chain and TCA cycle. Both the nicotinamide adenine dinucleotide (NADH) dehydrogenase activity of complex I (the first electron transporter in the electron transport chain) and the enzymatic activity of α-KGDH (which controls the metabolic flux of the TCA cycle) were decreased in the pWAT and eWAT of KKAy mice (Figures 4H,I,K,L). Empagliflozin markedly enhanced the activity of these two enzymes in the pWAT and eWAT of KKAy mice, which was likely the mechanism responsible for enhanced glucose metabolism and oxidative phosphorylation, which are the biochemical characteristics of metabolically active BAT.

Next, we investigated whether the alterations empagliflozin induced in browning and mitochondrial proteins were attributable to its glucose-lowering effect by administering insulin to KKAy mice. In contrast with empagliflozin, insulin treatment neither induced an increase in UCP1, PGC-1α, CYTO C, or MFN2, nor reduced the level of p-DRP1 (S616) in the pWAT and eWAT of KKAy mice (Supplementary Figures 2I–L). Therefore, the reduction of blood glucose alone did not account for the modulation of browning and mitochondrial dynamics by empagliflozin.

### Empagliflozin Induces a Brown-Like Phenotype and Stimulates Mitochondrial Biogenesis in Mouse Adipocytes

**FIGURE 5:**
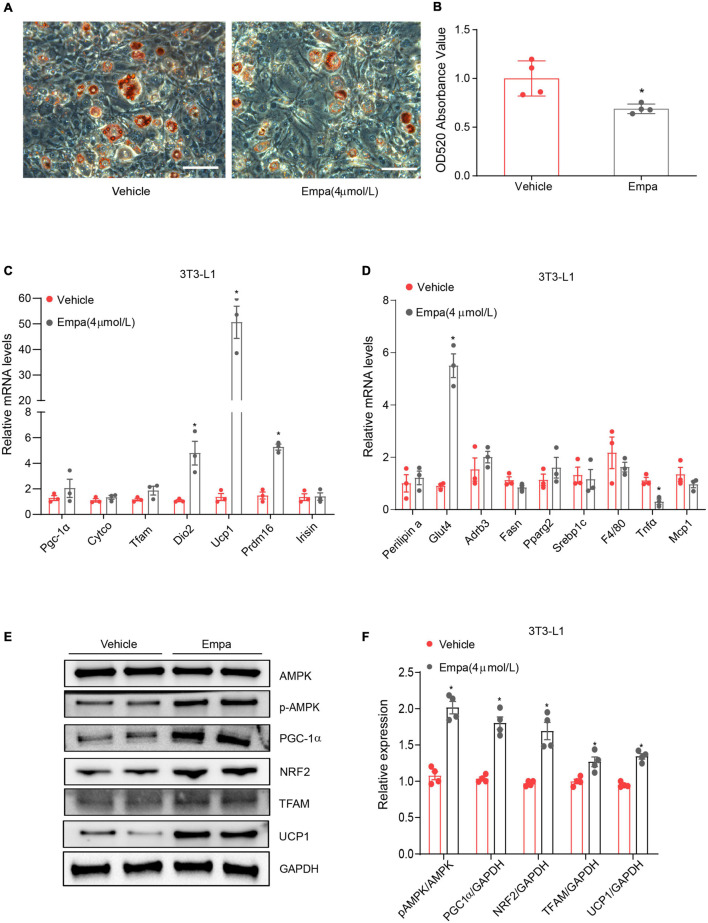
Empagliflozin induces a brown-like phenotype and elevates mitochondrial biogenesis in 3T3-L1 adipocytes. Differentiation of 3T3-L1 preadipocytes was induced through incubation in adipocyte-inducing medium for 5 days and then treatment (Empa) with or without (Vehicle) empagliflozin (4 μmol/L) in normal culture medium. **(A,B)** Oil Red O staining and quantification. Scale bar represents 100 μm. **(C,D)** qRT-PCR analysis of mRNAs of UCP1 and genes involved in mitochondrial biogenesis (*Pgc-1*α, *Cyto c*, *Tfam*, and *Dio2*), thermogenesis (*Prdm16* and *Irisin*), lipolysis (*Fasn* and *Srebf1*), lipogenesis (*Plin1*, *Adrb3*, and *Pparg*), and inflammation (*MCP1*, *Tnf*α, and *F4/80*), with GAPDH serving as a loading control. **(E,F)** Western blot and quantitation of AMPK, p-AMPK, PGC-1α, NRF2, TFAM, and UCP-1, with GAPDH used as a loading control. Data are presented as mean ± SEM. *P < 0.05 vs. Vehicle.

**FIGURE 6:**
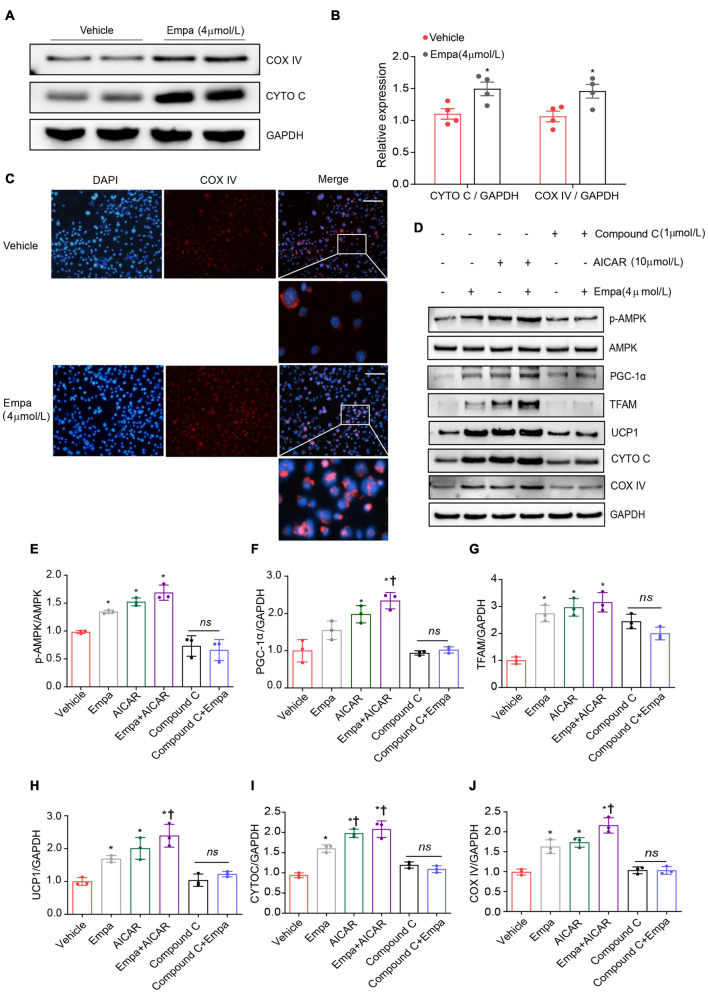
Upregulation of UCP1 and mitochondrial biogenesis by empagliflozin are dependent on AMPK activity. **(A,B)** Western blot and quantitation of COX IV and CYTO C with GAPDH as a loading control. **(C)** Immunostaining was used for analysis of the abundance of COX IV in empagliflozin-treated 3T3-L1 cells. COX IV was labeled red, and the nuclei of cells were stained blue. Representative images are shown. Image magnification is 200 ×. **(D–J)** Mature 3T3-L1 adipocytes were treated with (Empa) or without (Vehicle) empagliflozin (4 μmol/L), with or without AICAR (10 μmol/L), and with or without Compound C (1 μmol/L). Western blot and quantitation of p-AMPK, AMPK, PGC-1α, TFAM, UCP1, CYTO C, and COX IV, with GAPDH used as a loading control. Data are presented as mean ± SEM. *P < 0.05 vs. Vehicle; ^†^*P* < 0.05 vs. Empa.

Next, we investigated the browning effect of empagliflozin and its related mechanisms by establishing an *in vitro* model of mature adipocytes differentiated from 3T3-L1 preadipocytes. The mature 3T3-L1 adipocytes constitutively expressed SGLT2, which is the target protein of empagliflozin (Supplementary Figure 3A). Treatment with 4 μmol/L of empagliflozin for 72 h was chosen as the optimal condition on the basis of cell viability (Supplementary Figure 3B), lipid content (Supplementary Figure 3C), and COX IV expression (Supplementary Figures 3D–G). Empagliflozin changed the morphology of 3T3-L1 adipocytes, as evidenced by decreased Oil Red O staining, which displayed smaller lipid droplets and lower lipid content (Figures 5A,B). In line with the *in vivo* results, as evidenced by qRT-PCR, empagliflozin upregulated the expression of genes involved in the browning process, including *Ucp1*, *Prdm16*, and *Irisin*, as well as genes related to lipid/glucose metabolism and inflammation (Figures 5C,D). Furthermore, AMPK-PGC-1α signaling and the downstream transcription factors TFAM and NRF2 in 3T3-L1 adipocytes were upregulated by empagliflozin (Figures 5E,F). Empagliflozin also increased the mitochondrial biogenesis, as assessed by immunofluorescence and western blot (Figures 6A–C). Thus, empagliflozin induced white-to-brown conversion in mouse adipocytes and also increased mitochondrial biogenesis.

### Empagliflozin Induces Adipocyte Browning via the Adenosine Monophosphate–Activated Protein Kinase Signaling Pathway

Given the concurrent activation of AMPK signaling and adipocyte browning, we hypothesized that empagliflozin might induce the brown-like phenotype and mitochondrial biosynthesis in adipocytes via the AMPK pathway. This hypothesis was tested using the AMPK activator AICAR and its inhibitor Compound C. AICAR recapitulated the effects of empagliflozin in 3T3-L1 adipocytes, activating AMPK-PGC-1α-TFAM signaling and increasing the expression of UCP1 as well as that of the mitochondrial proteins CYTO C and COX IV (Figures 6D–J). In contrast, in the presence of Compound C, empagliflozin was unable to activate AMPK-PGC-1α-TFAM signaling or elevate the expression of UCP1 or mitochondrial proteins in adipocytes (Figures 6D–J). Therefore, it is likely that AMPK serves as a key signaling node in empagliflozin-induced browning and mitochondrial biogenesis.

### Empagliflozin Regulates Mitochondrial Homeostasis via Peroxisome Proliferator–Activated Receptor-γ Coactivator-1-alpha

**FIGURE 7:**
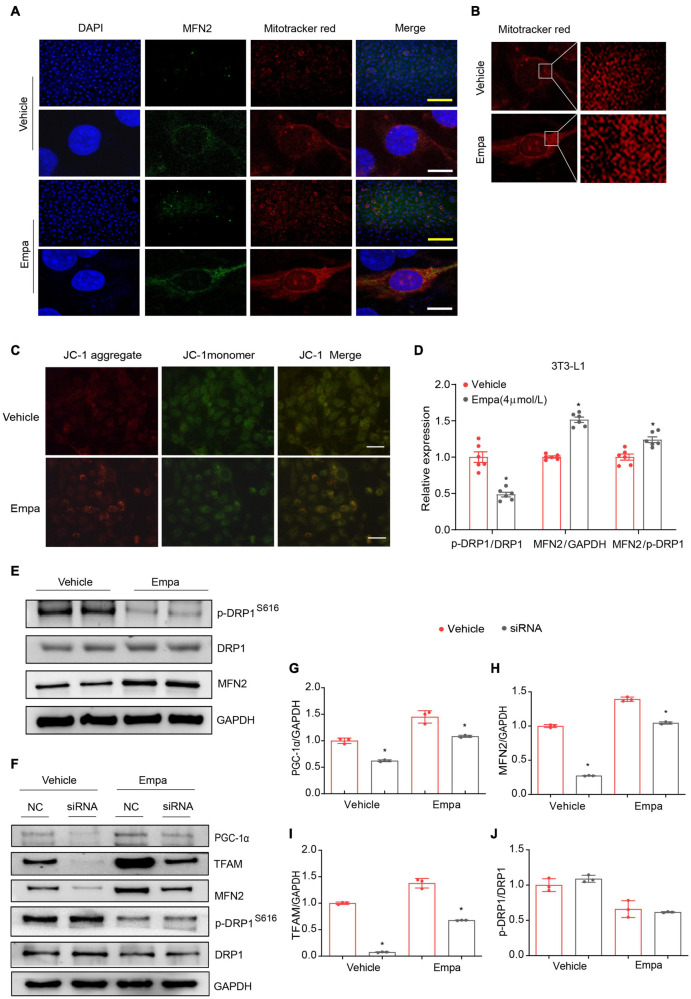
Empagliflozin induces mitochondrial fusion via PGC-1α and increases mitochondrial membrane potential in 3T3-L1 adipocytes. Mature 3T3-L1 adipocytes were treated with (Empa) or without (Vehicle) empagliflozin (4 μmol/L). **(A)** Immunofluorescence staining of mitochondria with MitoTracker Red (red) and of MFN2 (green) with nuclei stained blue with 4′,6-diamidino-2-phenylindole (DAPI); and **(B)** mitochondria stained with MitoTracker Red under confocal microscopy. Immunofluorescence scale bar represents 10 μm, Immunoconfocal scale bar represents 200 μm. **(C)** Analysis of mitochondrial membrane potential with JC-1. Representative images of JC-1 aggregates (red), monomers (green), and both aggregates and monomers. Scale bar represents 100 μm. **(D,E)** Western blot and quantitation of p-DRP1 (S616), DRP1, and MFN2, with GAPDH used as a loading control. **(F–J)** Mature 3T3-L1 adipocytes were transfected with negative control small interfering RNA (siRNA; NC) or *Pgc-1*α siRNA (siRNA) for 24 h and then treated with (Empa) or without (Vehicle) empagliflozin (4 μmol/L) for 72 h. Western blot and quantitation of PGC-1α, TFAM, MFN2, and DRP1, with GAPDH used as a loading control. Data are presented as the mean ± SEM of 3 independent experiments. *P < 0.05 vs. NC.

The *in vivo* results indicated empagliflozin to be a modulator of mitochondrial fusion and fission (Figures 4A–F). In the next part of our study, we evaluated mitochondrial morphological changes in 3T3-L1 adipocytes under empagliflozin treatment. Immunofluorescence and immunoconfocal microscopy revealed that the mitochondria in 3T3-L1 adipocytes were short and largely fragmented, and expressed low levels of MFN2 protein (Figures 7A,B). Comparatively, in empagliflozin-treated adipocytes, the mitochondria presented as elongated tubes with highly interconnected networks and an increased expression of MFN2 (Figures 7A,B). Since mitochondrial fusion contributes to the quality control of functional mitochondria, we performed JC-1 staining to evaluate mitochondrial membrane potential. Empagliflozin decreased JC-1 monomers while increasing JC-1 aggregates in 3T3-L1 adipocytes, which was indicative of hyperpolarization and enhanced mitochondrial integrity (Figure 7C). Further, empagliflozin increased the expression of mitochondrial respiratory chain complexes, as assessed by qRT-PCR and western blot (Supplementary Figures 4A,D,E). Empagliflozin also elevated the enzymatic activity of complex I and α-KGDH (Supplementary Figures 4B,C). Empagliflozin treatment improved mitochondrial quality, as it resulted in more energy-efficient and functional mitochondria, which might have met the energy requirements during browning.

Peroxisome proliferator–activated receptor-γ coactivator-1-alpha regulates mitochondrial fusion through modulating the expression of MFN2 (Lagouge et al., 2006). We carried out knockdown of *Pgc-1*α by transfecting 3T3-L1 adipocytes with *Pgc-1*α siRNA, and the efficiency of *Pgc-1*α knockdown was verified by decreases in PGC-1α and TFAM (Figures 7F,G,I) and no significant change in p-DRP1/DRP1 expression (Figure 7J). While empagliflozin increased MFN2 and concomitantly decreased p-DRP (S616) (Figures 7D,E), knockdown of *Pgc-1*α abolished the empagliflozin-induced upregulation of MFN2 (Figures 7F,H). These results suggested that PGC-1α might function as a critical regulator of MFN2 to initiate the mitochondrial fusion process following empagliflozin treatment.

## Discussion

Sodium-glucose co-transporter-2 inhibitors have received increasing attention as promising therapeutic agents for the treatment of metabolic diseases. Apart from having a primary glucose-lowering effect, SGLT2 inhibitors, such as empagliflozin, also reduce bodyweight and improve metabolic health in patients with T2DM (Häring et al., 2013; Chilton et al., 2015; Davidson and Sloan, 2017). Previous studies have shed light on possible mechanisms of the weight-loss effect of SGLT2 inhibitors, including increased urinary glucose excretion, WAT browning, and increased heat production in BAT (Xu et al., 2017; Xu and Ota, 2018). Consistent with the findings of previous reports, we demonstrated that empagliflozin reduced the bodyweight of mice in an *in vivo* model of T2DM, and this effect may have resulted from WAT browning. Using both *in vivo* and *in vitro* models, we found that the white-to-brown conversion induced by empagliflozin was accompanied by increased mitochondrial biogenesis, a shift toward mitochondrial fusion, and enhanced mitochondrial integrity and function. Furthermore, we showed that empagliflozin-induced browning and mitochondrial biogenesis might be mediated through the AMPK signaling pathway, while the induction of mitochondrial fusion by empagliflozin might be regulated via PGC-1α.

The imbalance between the energy-storing WAT and energy-dissipating BAT disrupts energy homeostasis, leaving the body predisposed to obesity and metabolic diseases (Bartelt and Heeren, 2014). A cluster of brown-like fat depots, defined as beige adipocytes, may be readily induced through WAT transformation under certain conditions or drug treatments. Beige adipocytes express the uncoupling protein UCP1 and display biochemical characteristics typical of BAT, such as abundance in mitochondria, high mitochondrial respiratory activity, and heat production (Wu et al., 2012; Ikeda et al., 2018). Chronic cold exposure, exercise, cyclic AMP, and PPARγ agonists are all examples of stimuli that can elicit the browning process (Wu et al., 2012; Ikeda et al., 2018), and scientists are actively pursuing other agents able to target this process to treat metabolic diseases. Our findings evidence empagliflozin to be a promising browning activator. Under cold exposure, empagliflozin-treated mice had a higher capacity for heat production, indicating the presence of thermogenic adipocytes. Specifically, in KKAy mice, in the perirenal and epididymal fat depots, which are characterized as WAT (Hilton et al., 2015), empagliflozin increased the levels of UCP1, remodeled the metabolic network, and promoted mitochondrial biogenesis and fusion, which are typical biochemical changes that occur during browning. Based on the expression of metabolic genes, empagliflozin could be seen to promote a shift from lipogenesis and inflammation toward glucose metabolism, lipolysis, and thermogenesis, which might have led to an increase in fatty acid oxidation and fat suppression. The conversion of WAT into BAT-like adipocytes in these depots conferred anti-obesity benefits to the mice, which manifested as substantial weight loss and decreased fat deposition. Our findings are consistent with those of previous studies reporting favorable metabolic phenotypes induced by empagliflozin in obese mice fed a high-fat diet, in which an increase in energy expenditure, elevated expression of UCP1 in WAT, attenuation of inflammation, and alleviation of hepatic steatosis were observed (Xu et al., 2017, 2019). It is currently unknown whether the browning transformation of WAT is a mechanism responsible for the effects of empagliflozin on weight loss and increased energy expenditure observed in humans in clinical trials, which may also result from increased urinary glucose excretion leading to mild osmotic diuresis and loss of calories in urine (Häring et al., 2013, 2014; Rosenstock et al., 2014).

Previous studies have identified AMPK as a regulator of BAT activation and WAT browning. AMPK-ablated mice were observed to show a deficit in non-shivering thermogenesis by BAT and browning of WAT, and also developed insulin resistance and non-alcoholic fatty liver disease (Mottillo et al., 2016; Desjardins and Steinberg, 2018). Conversely, in another study, an AMPK activator stimulated WAT browning, increased energy expenditure, and prevented obesity and metabolic abnormalities in mice fed a high-fat diet (Wu et al., 2018). Furthermore, AMPK modulates the activity of PGC1α to control the initiation of mitochondrial biogenesis, which is required for browning (Jäger et al., 2007). AMPK has also been recognized as a plausible mediator of the pleiotropic metabolic benefits of empagliflozin (Kim et al., 2018). Our study demonstrated that the effects of empagliflozin on UCP1 upregulation and mitochondrial biogenesis were dependent on AMPK activation. It can be presumed that after exposure to empagliflozin, activated AMPK mobilizes downstream targets to initiate the changes required for WAT browning, which include mitochondrial biogenesis and metabolic rewiring.

Under normal conditions, the number and quality of mitochondria are strictly controlled through biogenesis, degradation, fusion, and fission. A feature of BAT and beige adipocytes is the enrichment of mitochondrial cristae density, which enhances the metabolic activity of mitochondria in order to meet the high energy demand (Ikeda et al., 2018). Consistently, in this study, empagliflozin-induced browning of WAT was coupled with mitochondrial biogenesis, as well as altered mitochondrial gene expression and increased enzymatic activity. In parallel with *de novo* biogenesis, empagliflozin also tipped the fusion–fission balance toward fusion. The inhibition of fusion is well known to result in mitochondrial fragmentation and loss of membrane potential (Liesa and Shirihai, 2013), while increased fusion contributes to the maintenance of high-quality, functional mitochondria. Indeed, empagliflozin treatment results in the formation of elongated and interconnective mitochondria with high membrane potential. Disturbance of mitochondrial dynamics and homeostasis in adipocytes has been linked to a myriad of metabolic diseases (Liesa and Shirihai, 2013). Compared to individuals of a normal weight, individuals with obesity have fewer mitochondria, lower oxygen consumption, and impaired oxidative phosphorylation capacity in adipose tissue, which in turn propagates systemic obesity and metabolic disorders (Heinonen et al., 2015; Engin, 2017). Mitochondrial dysfunction in obesity leads to increased production of reactive oxygen species, which may result in inflammation and pathological changes in other organs (de Mello et al., 2018). As observed in the present study, the improvement of mitochondrial quality and function by empagliflozin may translate into multiple clinical benefits in patients with T2DM, including energy balance restoration, obesity prevention, and the promotion of systemic metabolic health.

Peroxisome proliferator–activated receptor-γ coactivator-1-alpha is recognized as a transcriptional regulator of MFN2, which functions by binding to the MFN2 promoter. The modulation of MFN2 fusion activity by PGC-1α is more likely to occur in energy-demanding rather than basal conditions (Arany et al., 2005; Liesa et al., 2008). MFN2 is expressed at a high level in BAT, and its function of mediating mitochondrial fusion is required for browning and related metabolic adaptation of mitochondria (Boutant et al., 2017). This study has provided preliminary evidence that PGC-1α is an indispensable regulator of mitochondrial fusion stimulated by empagliflozin. Further studies are needed to validate this mechanism and to explore its role in the browning effect of empagliflozin.

The hyperglycemic microenvironment has been reported to possibly drive a shift in mitochondrial dynamics to fusion, resulting in impaired mitochondrial integrity and function (Shenouda et al., 2011). However, our results show that insulin was unable to replicate the beneficial effects of empagliflozin on browning and mitochondrial biogenesis, thus indicating that these effects are not attributable to a reduction in blood glucose. Furthermore, insulin has been evidenced as being a suppressor of UCP1 expression and mitochondrial respiration in WAT and BAT *in vivo* (Dallon et al., 2018), which may contribute to weight gain in patients with T2DM receiving insulin therapy (Dallon et al., 2018). Therefore, empagliflozin may have an advantage over insulin in the treatment of T2DM, as it avoids the unwanted effect of weight gain and promotes weight loss.

Empagliflozin has also been shown to reduce cardiac injury and visceral adipocyte hypertrophy in prediabetic rats with metabolic syndrome (Kusaka et al., 2016). Moreover, compared with a placebo, empagliflozin improved cardio-renal outcomes in patients with T2DM with and without metabolic syndrome (Ferreira et al., 2020).

**FIGURE 8:**
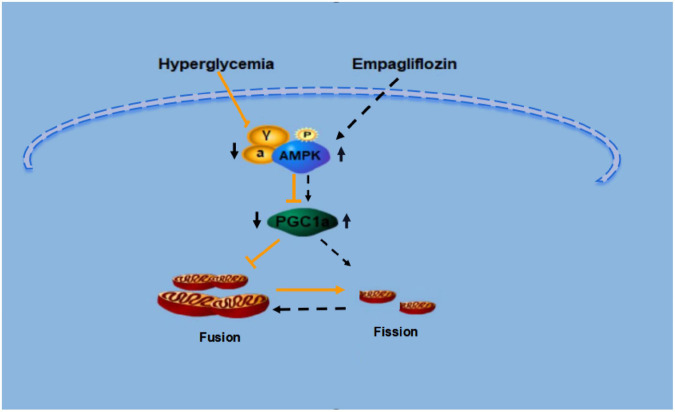
Proposed model for the signaling pathway by which empagliflozin induces white adipocyte browning and modulates mitochondrial dynamics via an AMPK dependent pathway.

In conclusion, our results demonstrate that empagliflozin is a promising anti-obesity agent that reduces bodyweight through the induction of WAT browning. Also, empagliflozin activates mitochondrial biogenesis and fusion, and improves mitochondrial function. The present study has also illustrated potential mechanisms underlying these effects; namely, that AMPK and PGC-1α may each mediate the regulation of browning and mitochondrial fusion under empagliflozin treatment. The detailed mechanisms of the regulation of WAT browning and mitochondrial dynamics by empagliflozin merit further investigation (Figure 8). Whether empagliflozin activates AMPK by inhibition of SGLT2 or by independent mechanisms remains to be tested. Besides, further clinical studies are also warranted to confirm the beneficial effects of empagliflozin on weight loss and metabolic homeostasis.

## Data Availability Statement

The original contributions presented in the study are included in the article/Supplementary Material, further inquiries can be directed to the corresponding author/s.

## Ethics Statement

The animal study was reviewed and approved by the Animal Ethics and Experimental Committee of the Metabolic Disease Hospital of Tianjin Medical University.

## Author Contributions

XLiu, XLi, TL, and XY performed the experiments and collected the data. MX, CX, DM, FS-G, and JY contributed to discussions. CK and NY critically reviewed and revised the manuscript. BS and LC contributed to the experimental design. LX wrote the manuscript. NB contributed to the manuscript preparation. All authors approved the final version of the manuscript.

## Conflict of Interest

The authors declare that the research was conducted in the absence of any commercial or financial relationships that could be construed as a potential conflict of interest.

## Publisher’s Note

All claims expressed in this article are solely those of the authors and do not necessarily represent those of their affiliated organizations, or those of the publisher, the editors and the reviewers. Any product that may be evaluated in this article, or claim that may be made by its manufacturer, is not guaranteed or endorsed by the publisher.

## Supplementary Material

Supplementary Figure 1 Bodyweight, blood glucose levels, and body temperature of KKAy and C57BL/6J mice. Eight-week-old male KKAy mice were treated with saline (DM), and C57BL/6J mice (NC) were used as a control (*n* = 6 per group) **(A)** Bodyweight of the mice during 8 weeks of treatment. **(B)** Changes in random blood glucose after 8 weeks of treatment, and **(C)** intraperitoneal glucose tolerance test (IPGTT) levels during 8 weeks of treatment. **(D)** Body temperature of the mice during a 4-h cold test at 4°C. **(E,F)** Representative images of hematoxylin staining in KKAy and C57BL/6J mice. Data are presented as the mean ± SEM. **P* < 0.05 vs. the NC group. pWAT, perirenal white adipose tissue; eWAT, epididymal white adipose tissue.

Supplementary Figure 2 Insulin does not induce the expression of UCP1 or proteins related to mitochondrial biogenesis, fusion, and fission in KKAy mice. **(A,B)** Representative images of hematoxylin staining in iWAT and sWAT. **(C,D)** Western blotting and quantitation of SGLT2 in different organs of 16-week-old C57BL/6J mice. **(E,F)** Western blot and quantitation of SGLT2 in different organs of 16-week-old KKAy mice. **(G,H)** Western blot and quantitation of SGLT2 in different fat depots in 16-week-old C57BL/6J mice. **(I–L)** Eight-week-old male KKAy mice (*n* = 6 per group) were treated with (DM + Empa) or without empagliflozin (DM), or treated with insulin (Ins). Western blot and quantitation of PGC-1α, p-DRP1 (S616), DRP1, MFN2, UCP1, and CYTO C, with GAPDH used as a loading control. Data are presented as the mean ± SEM. ^#^*P* < 0.05 vs. the DM group. pWAT, perirenal white adipose tissue; eWAT, epididymal white adipose tissue. iWAT, inguinal white adipose tissue; sWAT, subcutaneous white adipose tissue.

Supplementary Figure 3 Determination of empagliflozin treatment condition in 3T3-L1 adipocytes. **(A)** Western blot analysis of SGLT2 expression in 3T3-L1 adipocytes. HK2 as a negative control. **(B)** Cell viability of 3T3-L1 adipocytes treated with empagliflozin at different concentrations. Data are presented as the mean ± SEM of 3 independent experiments. **(C)** Oil red O staining of 3T3-L1 adipocytes treated with different concentrations of empagliflozin for different durations. Image magnification is 100 ×. **(D–G)** Western blot and quantitation of COX IV, with GAPDH used as a loading control in 3T3-L1 adipocytes treated with different concentrations of empagliflozin for different durations. Data are presented as the mean ± SEM. **P* < 0.05 vs. Vehicle.

Supplementary Figure 4 Empagliflozin stimulates mitochondrial functions in 3T3-L1 adipocytes. **(A)** qRT-PCR analysis of mRNAs of mitochondrial complex I (*Ndufab1*), II (*Sdhd*), III (*Cox7a1*), IV (*Cox8b*), and TCA cycle genes (*Cs*, *Idh3a*, and *Ogdh*), with GAPDH serving as a loading control. **(B,C)** Measurement of α-KGDH **(B)** and complex I enzymatic activity **(C)**. **(D,E)** Western blot and quantitation of mitochondrial respiratory chain complex I (NDUFA9), II (SDHA), and III (CYTB), with GAPDH used as a loading control. Data are presented as the mean ± SEM. **P* < 0.05 vs. Vehicle.

## Abbreviations

BAT: brown adipose tissue
COX IV: cytochrome C oxidase subunit IV
CYTO C: cytochrome C
DRP1: dynamin-related protein 1
ETC: electron transport chain
eWAT: epididymal white adipose tissue
MFN1: mitofusin 1
MFN2: mitofusin 2
NADH: nicotinamide adenine dinucleotide
NRF2: nuclear factor erythroid 2–related factor 2
PGC1 α: peroxisome proliferator–activated receptor γ -coactivator factor-1
pWAT: perirenal white adipose tissue
SGLT2: sodium-glucose co-transporter-2
TCA: tricarboxylic acid
TFAM, transcription factor A: mitochondrial
UCP1: uncoupling protein 1
WAT: white adipose tissue
α -KGDH: alpha-ketoglutarate dehydrogenase.
