# Supplementary figures and images for: Empagliflozin Induces White Adipocyte Browning and Modulates Mitochondrial Dynamics in KK Cg-Ay/J Mice and Mouse Adipocytes
Source: Front Physiol. 2021 Oct 27;12:745058. doi: 10.3389/fphys.2021.745058 (PMC8578598; doi:10.3389/fphys.2021.745058)

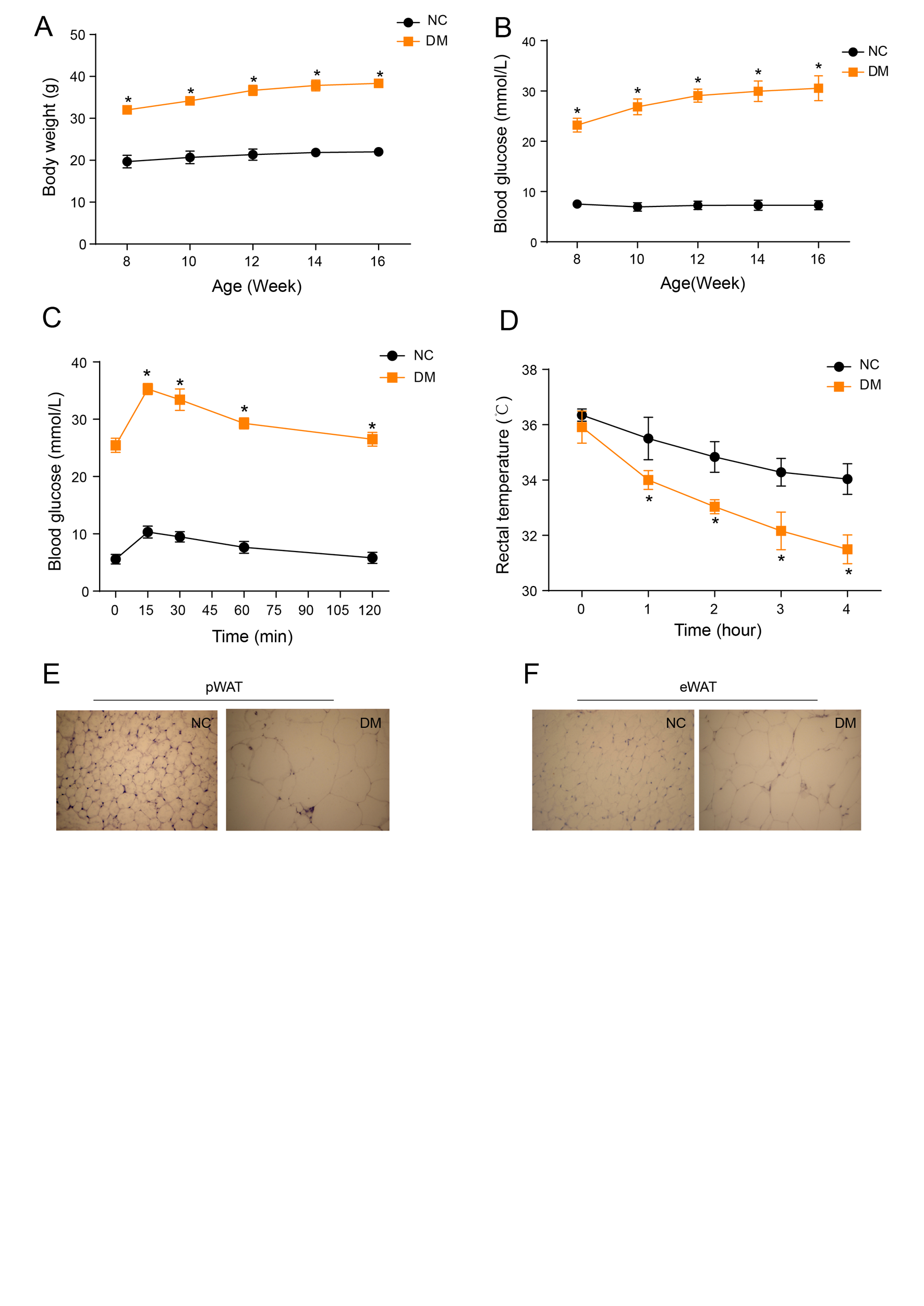

Supplement: Supplementary Figure 1 — Bodyweight, blood glucose levels, and body temperature of KKAy and C57BL/6J mice. Eight-week-old male KKAy mice were treated with saline (DM), and C57BL/6J mice (NC) were used as a control (n = 6 per group) (A) Bodyweight of the mice during 8 weeks of treatment. (B) Changes in random blood glucose after 8 weeks of treatment, and (C) intraperitoneal glucose tolerance test (IPGTT) levels during 8 weeks of treatment. (D) Body temperature of the mice during a 4-h cold test at 4°C. (E,F) Representative images of hematoxylin staining in KKAy and C57BL/6J mice. Data are presented as the mean ± SEM. *P < 0.05 vs. the NC group. pWAT, perirenal white adipose tissue; eWAT, epididymal white adipose tissue. [file Image_1.TIF]

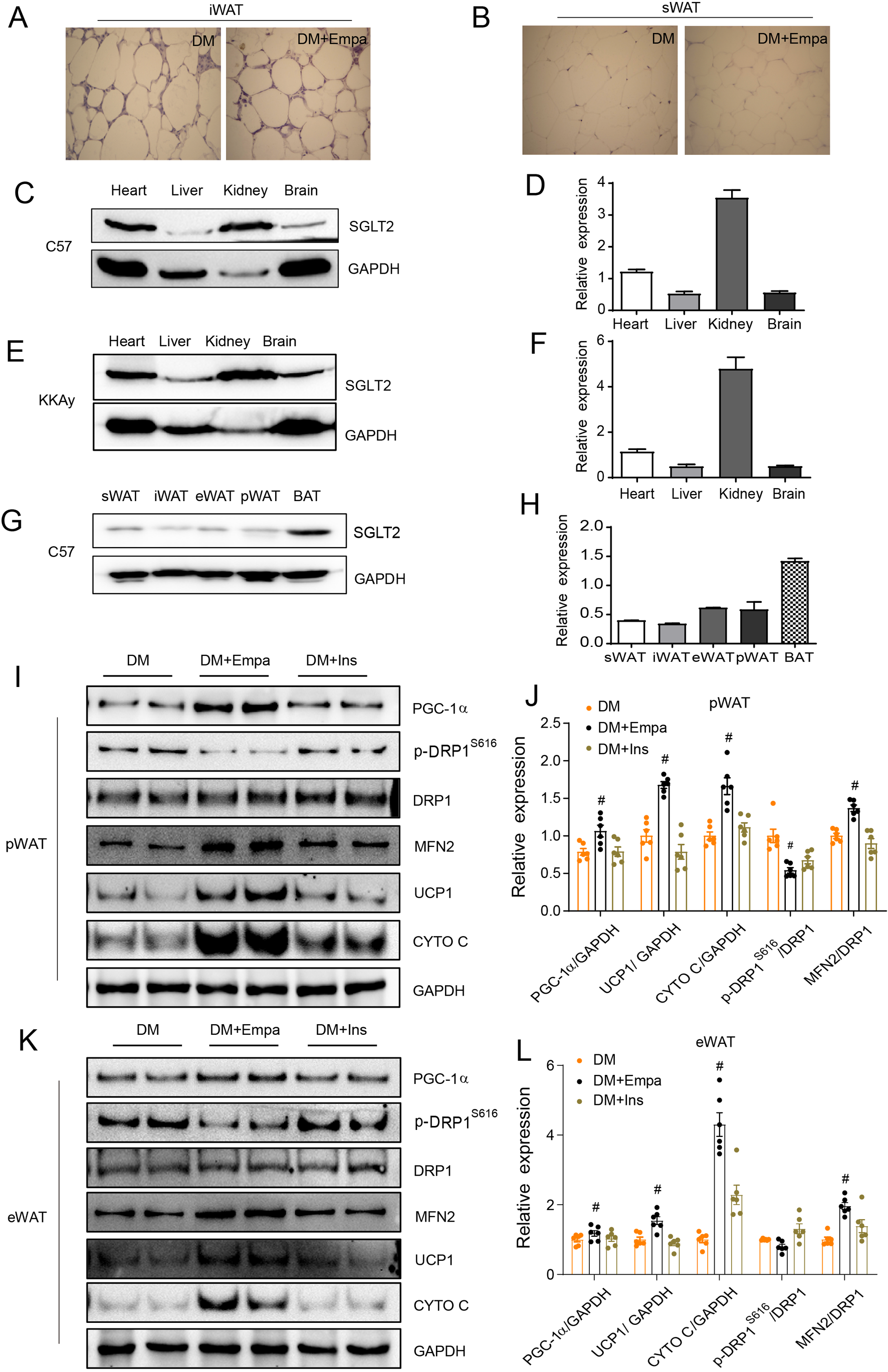

Supplement: Supplementary Figure 2 — Insulin does not induce the expression of UCP1 or proteins related to mitochondrial biogenesis, fusion, and fission in KKAy mice. (A,B) Representative images of hematoxylin staining in iWAT and sWAT. (C,D) Western blotting and quantitation of SGLT2 in different organs of 16-week-old C57BL/6J mice. (E,F) Western blot and quantitation of SGLT2 in different organs of 16-week-old KKAy mice. (G,H) Western blot and quantitation of SGLT2 in different fat depots in 16-week-old C57BL/6J mice. (I–L) Eight-week-old male KKAy mice (n = 6 per group) were treated with (DM + Empa) or without empagliflozin (DM), or treated with insulin (Ins). Western blot and quantitation of PGC-1α, p-DRP1 (S616), DRP1, MFN2, UCP1, and CYTO C, with GAPDH used as a loading control. Data are presented as the mean ± SEM. #P < 0.05 vs. the DM group. pWAT, perirenal white adipose tissue; eWAT, epididymal white adipose tissue. iWAT, inguinal white adipose tissue; sWAT, subcutaneous white adipose tissue. [file Image_2.TIF]

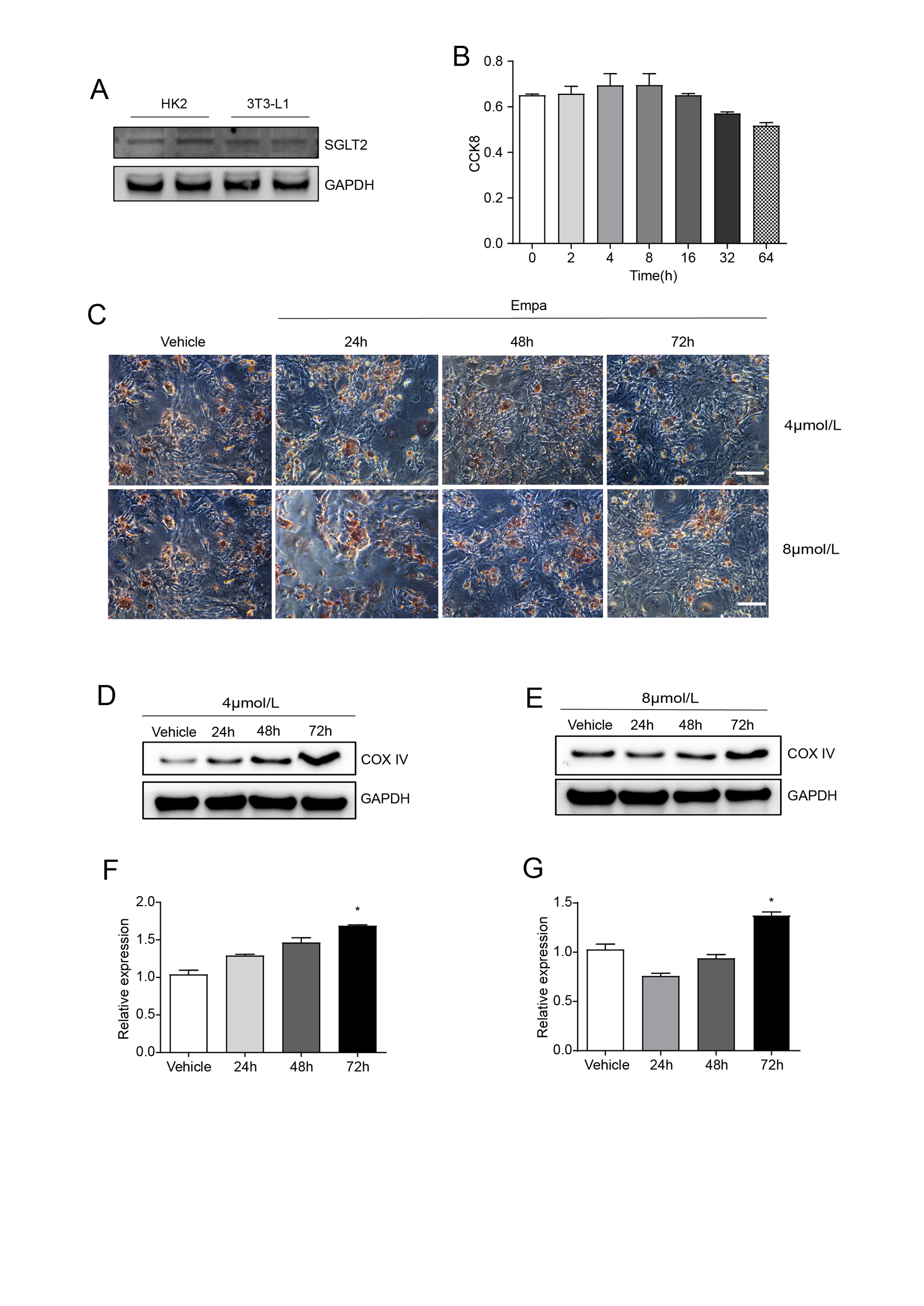

Supplement: Supplementary Figure 3 — Determination of empagliflozin treatment condition in 3T3-L1 adipocytes. (A) Western blot analysis of SGLT2 expression in 3T3-L1 adipocytes. HK2 as a negative control. (B) Cell viability of 3T3-L1 adipocytes treated with empagliflozin at different concentrations. Data are presented as the mean ± SEM of 3 independent experiments. (C) Oil red O staining of 3T3-L1 adipocytes treated with different concentrations of empagliflozin for different durations. Image magnification is 100 ×. (D–G) Western blot and quantitation of COX IV, with GAPDH used as a loading control in 3T3-L1 adipocytes treated with different concentrations of empagliflozin for different durations. Data are presented as the mean ± SEM. *P < 0.05 vs. Vehicle. [file Image_3.TIF]

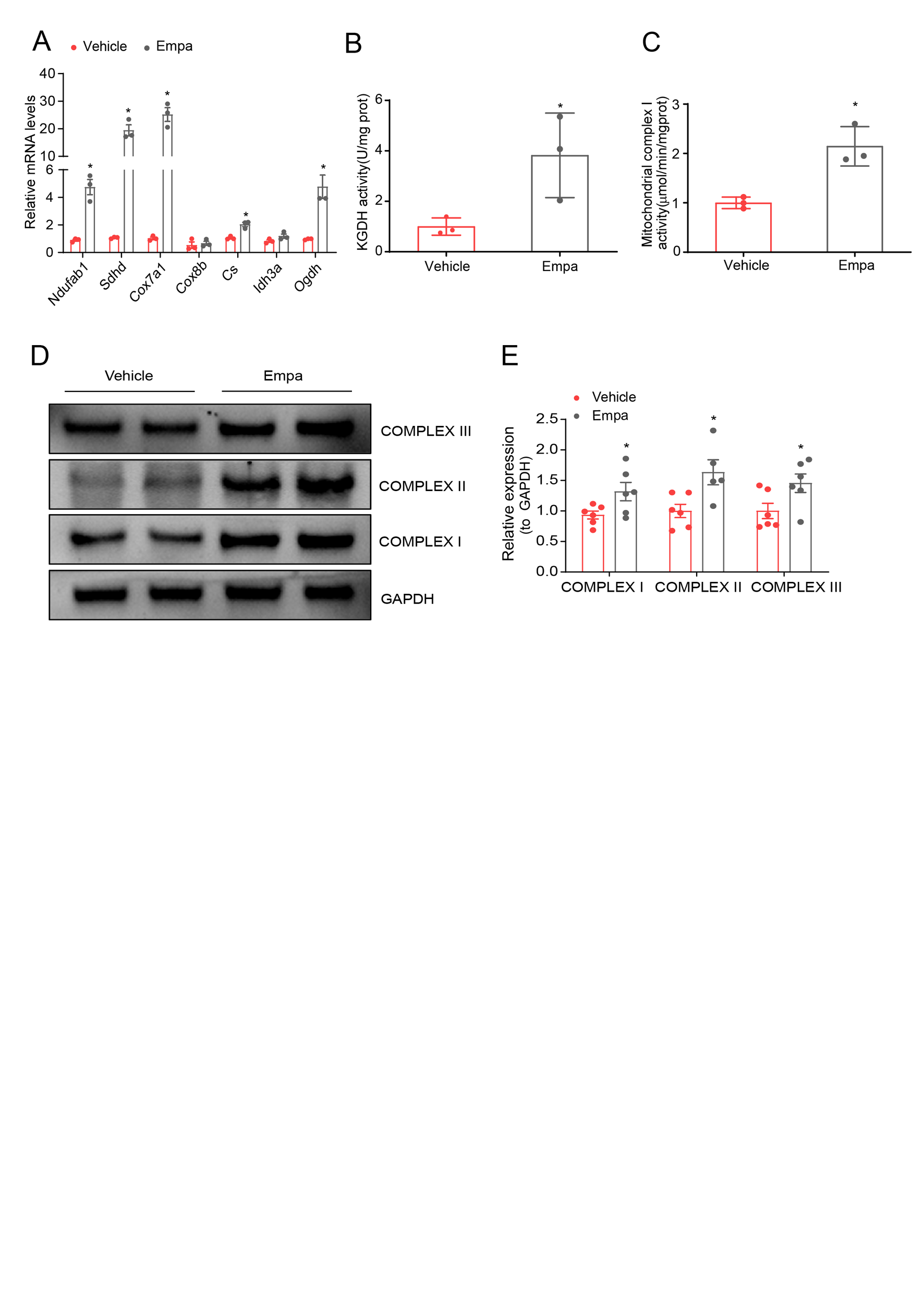

Supplement: Supplementary Figure 4 — Empagliflozin stimulates mitochondrial functions in 3T3-L1 adipocytes. (A) qRT-PCR analysis of mRNAs of mitochondrial complex I (Ndufab1), II (Sdhd), III (Cox7a1), IV (Cox8b), and TCA cycle genes (Cs, Idh3a, and Ogdh), with GAPDH serving as a loading control. (B,C) Measurement of α-KGDH (B) and complex I enzymatic activity (C). (D,E) Western blot and quantitation of mitochondrial respiratory chain complex I (NDUFA9), II (SDHA), and III (CYTB), with GAPDH used as a loading control. Data are presented as the mean ± SEM. *P < 0.05 vs. Vehicle. [file Image_4.TIF]
