# Supplementary material for: Empagliflozin Induces White Adipocyte Browning and Modulates Mitochondrial Dynamics in KK Cg-Ay/J Mice and Mouse Adipocytes
Source: Front Physiol. 2021 Oct 27;12:745058. doi: 10.3389/fphys.2021.745058 (PMC8578598; doi:10.3389/fphys.2021.745058)
Supplement: Supplementary file 5 [file Data_Sheet_1.pdf]

Supplementary Tables

Supplementary Table 1. Primary antibodies used for Western blot analysis

Antibody	Manufacturer	Dilution factor	Catalogue number
AMPK	Abcam, USA	1:1000	ab207442
p-AMPK	Abcam, USA	1:1000	ab133448
PGC-1α	Proteintech, China	1:1000	20658-1-AP
NRF2	Abcam, USA	1:1000	ab76026
UCP1	Proteintech, China	1:500	23673-1-AP
TFAM	Abcam, USA	1:500	ab131607
COX IV	Proteintech, China	1:4000	11242-1-AP
CYTO C	Proteintech, China	1:2000	10993-1-AP
DRP1	Cell Signaling Technology, USA	1:1000	#8570
p-DRP1	Cell Signaling Technology, USA	1:1000	#3455
MFN2	Cell Signaling Technology, USA	1:1000	#9482
Complex I	Proteintech, China	1:1000	20312-1-AP
Complex II	Proteintech, China	1:1000	14865-1-AP
Complex III	Proteintech, China	1:1000	55090-1-AP
SGLT2	Abcam, USA	1:1000	ab37296
GAPDH	Bioworld, USA	1:5000	AP0063

AMPK, adenosine 5′ monophosphate-activated protein kinase; PGC-1 α, peroxisome proliferator-activated receptor γ-coactivator factor-1; NRF2, nuclear factor erythroid 2-related factor 2; UCP1, uncoupling protein 1; TFAM, transcription factor A; COX IV, cytochrome C oxidase subunit IV; CYTO C, cytochrome C; DRP1, dynamin-related protein 1; MFN2, mitofusin-2; GAPDH, glyceraldehyde 3-phosphate dehydrogenase；SGLT2, Sodium-glucose co-transporter-2;

Supplementary Table 2. Sequences of primers used for RT-qPCR

Genes	Forward (5'-3')	Reverse (5'-3')
Plin1	GTCCGTCGGCTGTCCACCC	ATGCTGTTCCTGGCGCTTCG
Adrb3	CAGGTTTGATGGCTATGAAGG	GGGCAACCAGTCAAGAAGATG
Glut4	GGAGGGAGCCTTTGGTATTT	CAGCACAGGACACTCATCTT
Fasn	TGTGGTGGGTTTGGTGAATTGT	ACGAGGTCATGCTTTAGCACC
Pparg2	TCGCTGATGCACTGCCTATG	GAGAGGTCCACAGAGCTGATT
Srebf1	CCTCTGATCTCATGGCTCATAAC	CTAGGGAACTGTGTGTGTTTCT
Pgc-1α	GACAATCCCGAAGACACTACAG	AGAGAGGAGAGAGAGAGAGAGA
Ucp1	CTGCCAGGACAGTACCCAAG	TCAGCTGTTCAAAGCACACAAA
Cox8b	GCGAAGTTCACAGTGGTTCC	GGAACCATGAAGCCAACGACT
Dio2	GCTGCCGAATGTCAACGAATG	CTGGTTCAGACTCACCTTGGA
Cytoc	GCTACCCATGGTCTCATCGTG	CATCATCATTAGGGCCATCCT
Tfam	GTCCATAGGCACCGTATTGCG	CCCATGCTGGAAAAACACTTCG
Mcp1	CCACTCACCTGCTGCTACTCAT	TGGTGATCCTCTTGTAGCTCTC
Tnf	GCCTCTTCTCATTCCTGCTTGT	GGCCATTTGGGAACTTCTCAT
F4/80	CGTCAGGTACGGGATGAATATAAG	ATCTTGGAAGTGGATGGCATAG
Ndufab1	GGCTGCTGACTGGAACTTACT	CTAGGCCAGATGTGTGGGAG
Ndufa8	GAGTTTATGCTGTGCCGCTG	TACTCTGTGAAAGGCTCCGC
Cox7a1	AGTACACTTGAAAGGCGGGG	GCCCAGCCCAAGCAGTATAA
Uqcrc2	TCCGACCCCATCTTGCTTTG	CGGGGCAACTTTGAGGGAAT
Atp5a1	CATTTTGTGCCAGTCGTCCG	CAGCTGTGCCAGTCTTCTGA
Idh3a	GAGTACGCTCGGAACAACCA	AGTTCTCCGCAACTTCCCTG
Cs	TGTTTCAGGGGCCTTTAAGACT	CATGCTTCAGTCCCGGTCAT
Ogdh	CCTGGGGATTTTGGATGCTGAT	GGCCATAGAACCCTCCTACTG
Gapdh	CATCACTGCCACCCAGAAGACTG	ATGCCAGTGAGCTTCCCGTTCAG

Plin1, perilipin1; *Adrb3*,beta3-adrenoceptor; *Glut4*, glucose transporter type 4; *Fasn,* fatty acid synthase; *Pparg2,* peroxisome proliferator-activated receptor 2; *Srebf1,* sterol regulatory element binding protein; *Pgc-1α,* peroxisome proliferator-activated receptor γ coactivator 1α; *Ucp1,* uncoupling protein 1; *Cox8b,* cytochrome c oxidase subunit 8B; *Dio2,* iodothyronine deiodinase 2; *Cytoc,* cytochrome c; *Tfam,* mitochondrial transcription factor A; *Mcp1,* monocyte chemotactic protein-1; *Tnfα,* tumor necrosis factor *α*; *F4/80,* F4/80 antigen; *Ndufab1,* NADH:Ubiquinone oxidoreductase subunit AB1; *Ndufa8,* NADH:Ubiquinone oxidoreductase subunit A8; *Cox7a1,* cytochrome c oxidase polypeptide 7A1; *Uqcrc2,* cytochrome b-c1 complex subunit 2; *Atp5a1,* ATP synthase F1 subunit α; *Cs,* citrate synthase; *Ogdh,* oxoglutarate dehydrogenase; *Gapdh,* glyceraldehyde 3-phosphate dehydrogenase
